# CRISPR in Neurodegenerative Diseases Treatment: An Alternative Approach to Current Therapies

**DOI:** 10.3390/genes16080850

**Published:** 2025-07-22

**Authors:** Amna Akbar, Rida Haider, Luisa Agnello, Bushra Noor, Nida Maqsood, Fatima Atif, Wajeeha Ali, Marcello Ciaccio, Hira Tariq

**Affiliations:** 1 Institute of Molecular Biology and Biotechnology, Bahauddin Zakariya University, Bosan Road, Multan 60800, Punjab, Pakistan; amanakbar818@gmail.com; 2 Department of Precision Medicine in Medical, Surgical, and Critical Care (Me.Pre.C.C.), University of Palermo, 90100 Palermo, Italy; rida.haider@unipa.it; 3 Institute of Clinical Biochemistry, Clinical Molecular Medicine, and Clinical Laboratory Medicine, Department of Biomedicine, Neurosciences, and Advanced Diagnostics (BIND), University of Palermo, via del Vespro, 129, 90127 Palermo, Italy; luisa.agnello@unipa.it; 4 Department of Pharmacology, Faculty of Pharmacy and Pharmaceutical Sciences, University of Karachi, Karachi 75270, Sindh, Pakistan; bushnoor700@gmail.com; 5 Department of Biotechnology, University of Sargodha, University Road, Sargodha 40100, Punjab, Pakistan; nidamaqsood768@gmail.com (N.M.); atiffatima407@gmail.com (F.A.); wajeeha.ali@gmail.com (W.A.); 6 Department of Laboratory Medicine, University Hospital “P. Giaccone”, Via del Vespro, 129, 90127 Palermo, Italy; 7 Institute of Microbiology and Molecular Genetics, University of the Punjab, Quaid-i-Azam Campus, Lahore 54590, Punjab, Pakistan; hiratariq390@gmail.com

**Keywords:** CRISPR-Cas9, neurodegenerative diseases, therapy, gene, Alzheimer’s disease, treatment

## Abstract

Neurodegenerative diseases (NDs) pose a major challenge to global healthcare systems owing to their devastating effects and limited treatment options. These disorders are characterized by progressive loss of neuronal structure and function, resulting in cognitive and motor impairments. Current therapies primarily focus on symptom management rather than on targeting the underlying causes. However, clustered regularly interspaced short palindromic repeat (CRISPR) technology offers a promising alternative by enabling precise genetic modifications that could halt or even reverse ND progression. CRISPR-Cas9, the most widely used CRISPR system, acts as a molecular scissor targeting specific DNA sequences for editing. By designing guide RNAs (gRNAs) to match sequences in genes associated with NDs, researchers can leverage CRISPR to knockout harmful genes, correct mutations, or insert protective genes. This review explores the potential of CRISPR-based therapies in comparison with traditional treatments for NDs. As research advances, CRISPR has the potential to revolutionize ND treatment by addressing its genetic underpinnings. Ongoing clinical trials and preclinical studies continue to expand our understanding and application of this powerful tool to fight debilitating conditions.

## 1. Introduction

Neurodegenerative diseases (NDs) such as Alzheimer’s disease (AD), Parkinson’s disease (PD), Huntington’s disease (HD), and amyotrophic lateral sclerosis (ALS) are a growing public health concern. These disorders result from gradual deterioration of the nervous system, leading to cognitive impairment and motor dysfunction [1]. As the global population ages, the prevalence and impact of NDs are expected to rise significantly, imposing substantial burdens on individuals, families, healthcare systems, and economies [2]. Consequently, there is an urgent need to develop more effective treatment strategies. Current therapeutic approaches primarily focus on symptomatic relief and slowing disease progression but fail to address the underlying causes [3]. For instance, HD and ALS rely on symptomatic treatments such as tetrabenazine for chorea and riluzole for ALS, both of which offer neuroprotective benefits. However, these treatments are limited by their inability to halt or reverse neuronal degeneration. Moreover, their efficacy often diminishes over time, and adverse side effects frequently accompany their use. The absence of disease-modifying therapies highlights a critical gap in ND management [4].

In recent years, significant advancements in genomic technology have transformed the ND research. One of the most promising genome-editing approaches is Clustered Regularly Interspaced Short Palindromic Repeats (CRISPR), which enables precise genetic modifications, including the correction of disease-causing mutations, regulation of gene expression, and introduction of protective genes. Although conventional therapies address the downstream effects of genetic alterations, CRISPR directly targets the molecular basis of these disorders [5].

This paradigm shift toward CRISPR-based therapies is driven by both the limitations of traditional treatments and an increasing understanding of the genetic mechanisms underlying NDs. Many NDs are strongly influenced by specific genetic mutations, such as *HTT* in HD; *APP*, *PSEN1*, *PSEN2*, and *SNCA* in familial forms of AD and PD; and *SOD1*, *C9orf72*, *TARDBP*, and *FUS* in ALS. CRISPR’s ability to target these mutations with unprecedented precision offers a revolutionary approach to ND treatment. CRISPR technology extends beyond gene editing to include gene activation, repression, and epigenetic modification. Innovations in delivery systems, such as viral vectors and nanoparticles, have further enhanced the potential of efficient and targeted brain therapies. These developments position CRISPR not as an incremental advancement but as a transformative step toward personalized medicine [6].

However, the transition from traditional therapies to CRISPR-based approaches is challenging. Issues related to delivery methods, specificity, potential off-target effects, and ethical considerations surrounding germline modifications must be carefully addressed [7].

This review explores the emerging role of CRISPR in the treatment of ND. We discuss current therapeutic strategies and their limitations, followed by an in-depth analysis of CRISPR technology, its challenges and ethical implications, and its potential to revolutionize ND management. While several reviews have examined the role of CRISPR technology in neurodegenerative diseases, the present manuscript offers a comprehensive and updated analysis that distinguishes itself in multiple ways. First, we integrate a broad spectrum of CRISPR modalities, including CRISPR-Cas9, base editing, prime editing, CRISPR interference, and CRISPR activation, providing a detailed comparison of their mechanisms, advantages, and limitations in the context of ND therapy. Second, unlike many previous reviews that focus exclusively on neuronal targets, we emphasize the therapeutic potential of CRISPR-based editing in glial cells, particularly astrocytes and microglia, which are increasingly recognized as key contributors to ND progression. This dual focus provides a more complete view of CRISPR’s role in modulating central nervous system pathology. Third, we include a side-by-side comparison of conventional treatments and CRISPR-based interventions, highlighting how genome editing offers a shift from symptomatic management to potentially disease-modifying strategies. Fourth, we incorporate and critically evaluate recent preclinical studies and advancements in in vivo delivery technologies, such as nanoparticles, as well as the latest insights into off-target effects and germline risks, supported by current animal data. Finally, we address ethical and translational considerations, including regulatory barriers and long-term safety, providing a realistic assessment of the path to clinical application.

Through these contributions, this review aims to serve as both an informative resource and a forward-looking framework for researchers and clinicians exploring CRISPR technologies in the treatment of neurodegenerative diseases.

## 2. Current Treatment of Neurodegenerative Diseases

Current ND therapies aim to manage symptoms, slow disease progression, and improve the quality of life of patients [8].

### 2.1. Alzheimer’s Disease

AD is a progressive ND characterized by cognitive decline, memory loss, and impaired daily functioning. Current treatments are primarily symptomatic, with little impact on disease causation [9].

Cholinesterase inhibitors (e.g., donepezil, rivastigmine, and galantamine) temporarily enhance cognitive function by preventing degradation of acetylcholine, a key neurotransmitter for memory and learning. However, these drugs provide only modest benefits and are associated with side effects such as nausea, vomiting, and diarrhea [10].

Memantine, an NMDA receptor antagonist, modulates glutamate activity to reduce excitotoxicity, a key driver of neuronal loss in AD. Although beneficial in moderate-to-severe AD, it does not halt disease progression [10].

Recently, monoclonal antibodies targeting amyloid-beta plaques, such as lecanemab, have been developed. Lecanemab, co-developed by Eisai Co., Ltd. and Biogen Inc., was approved by the FDA in 2023 after clinical trials demonstrated its ability to slow cognitive decline by 27% over 18 months [11]. However, lecanemab is associated with risks, including Amyloid-Related Imaging Abnormalities (ARIAs) and infusion-related reactions (e.g., fever, chills, and headache). Additionally, its high cost ($26,500 annually) presents an accessibility challenge [12,13].

Non-pharmacological interventions such as cognitive stimulation therapy, reminiscence therapy, and nutritional supplements (e.g., Ginkgo biloba) have been explored, although their efficacy remains inconclusive [14].

### 2.2. Parkinson’s Disease

PD is a progressive ND characterized by motor dysfunction due to the loss of dopamine-producing neurons in the substantia nigra (SN). The symptoms include bradykinesia, tremors, rigidity, postural instability, and cognitive and psychiatric complications.

Levodopa, a dopamine precursor, remains the gold standard therapy, often combined with carbidopa, for reducing peripheral degradation and enhancing its effectiveness. However, long-term use leads to motor complications, such as dyskinesia (involuntary movements) and fluctuations in drug efficacy [15]. Dopamine agonists (e.g., pramipexole) directly stimulate dopamine receptors and are often used to delay levodopa initiation [16]. MAO-B inhibitors (e.g., rasagiline and selegiline) prolong dopamine activity by inhibiting its breakdown and offering mild symptomatic relief [17].

Notably, current treatments do not reverse or slow PD progression, highlighting the need for disease-modifying therapies [18].

In 2018, the safety of transplantation of dopaminergic progenitor cells created using iPSCs into patients with Parkinson’s disease was evaluated in a clinical trial. The objective of this study was to re-establish motor function and restore lost dopamine-producing brain cells. The study was conducted at Kyoto University Hospital. The process included stereotactic implantation of approximately 2.4 million dopaminergic progenitor cells derived from iPSCs into the putamen, a PD-critical area. The cells were obtained from third-party donors and processed according to strict clinical-grade procedures to reduce risks, including tumor formation [19]. Tacrolimus was administered to reduce the risk of immune rejection. Adverse effects and graft survival indicators were also assessed. Preliminary data showed no apparent safety complications, cell survival, or integration indicators [20].

### 2.3. Huntington’s Disease

HD is a progressive neurodegenerative disorder caused by a mutation in the *HTT* gene, which leads to motor dysfunction, cognitive decline, and psychiatric symptoms. Although there is currently no cure, various therapeutic approaches aim to manage the symptoms, slow disease progression, and improve the quality of life of patients.

Current therapies attempt to counteract the motor and psychiatric symptoms to improve the patient’s condition. Tetrabenazine is one of the first drugs for managing chorea, jerky, and involuntary motions. Tetrabenazine inhibits vesicular monoamine transporter 2 (*VMAT2*), decreasing dopamine in the brain and inhibiting excessive motion. Tetrabenazine, however, does not inhibit disease progression and is accompanied by considerable side effects, including sedation, depression, and heightened susceptibility to suicidal ideation, and must therefore be monitored closely [21]. Other drugs, such as deutetrabenazine, a tetrabenazine analog, have been introduced to achieve similar therapeutic goals while limiting side effects [21].

Clinical trials comparing tetrabenazine to deutetrabenazine for Huntington’s disease (HD) were mainly conducted in North America and Europe, with central studies coordinated by the Huntington Study Group [22]. Tetrabenazine was assessed in a double-blind, randomized, placebo-controlled trial in 84 patients at several sites, where it decreased chorea scores assessed by means of the Unified Huntington’s Disease Rating Scale (UHDRS) significantly, although with side effects such as depression, somnolence, and akathisia [23].

Deutetrabenazine was subsequently tried in the first HD trial, a multicenter, double-blind, placebo-randomized trial of 90 HD patients. It leads to a significant reduction in chorea and offers improved tolerability due to its deuterated chemical structure and slower metabolic rate [24]. The two medications were taken orally in titrated form for several weeks, with a chorea clinical benefit seen within one month, although each did not change the course of the underlying disease. The trials reinforced the utility of medication in symptom control but made it even clearer how little impact treatments currently have on the course of the disease [25].

Additionally, antidepressants and antipsychotics are used to manage psychiatric symptoms such as depression, anxiety, aggressiveness, and irritability [26]. Selective serotonin reuptake inhibitor, such as fluoxetine and sertraline, and atypical antipsychotics such as risperidone and olanzapine are most used to improve depression and anxiety and to control aggressivity, paranoia, and hallucinations, respectively [27]. Although such drugs can improve a patient’s social function and emotional state, nothing can hinder the neurodegenerative process. Most of these drugs have additional side effects such as weight gain, sleepiness, and derangement in metabolic dysfunction, and long-term use can make them cumbersome [28].

### 2.4. Amyotrophic Lateral Sclerosis

ALS is a progressive neurodegenerative disorder that affects motor neurons. This leads to muscle weakness, atrophy, and paralysis. Although there is currently no cure, various therapeutic approaches involve disease progression and symptomatic care. The most important disease-altering therapies for ALS involve riluzole and edaravone [29]. Riluzole, a glutamate inhibitor, slows disease progression and prolongs survival by a few months [30]. Edaravone is an antioxidant that may reduce oxidative stress and slow functional decline in early-stage ALS. Although these drugs may slow the progression of ALS to some extent, overall patient survival remains poor, with life expectancy typically extended by only a few months [31].

Supportive therapies play a significant role in maintaining acceptable quality of life. Physical therapy maintains mobility, reduces joint stiffness, and prevents contracture. Adaptive equipment, such as braces and wheelchairs, aids movement. Speech and Swallowing Therapy addresses dysarthria (speech difficulties), provides alternative communication devices (e.g., speech-generating devices), and manages dysphagia (swallowing difficulties) to reduce the risk of aspiration pneumonia. Respiratory therapy monitors the lung function and provides breathing exercises. Tracheostomy and invasive ventilation may be required in advanced stages. Finally, nutritional therapies, such as high-calorie diets, are recommended because of the increased metabolic demands. Percutaneous endoscopic gastrostomy (PEG) feeding is used in patients with severe dysphagia [32,33]. Therapy for ALS is multidisciplinary and aims to slow its progression, manage symptoms, and maintain quality of life. While current treatments focus on symptom control and supportive care, ongoing research on disease-modifying therapies offers hope for future advancement.

Clinical studies of riluzole and edaravone were tested in randomized, double-blind, controlled studies in patients with early-stage ALS, where it was found to produce a survival benefit of approximately 2–3 months by inhibiting glutamate-induced excitotoxicity, one of the hypothesized pathogenetic mechanisms in ALS [34].

Edaravone was administered orally at a dose of 50 mg twice daily, and survival rates were monitored along with respiratory function. It has also been tested in a comparably controlled environment, mainly in Japan, in patients with early fast-progressing ALS. It was administered intravenously over 28 days (14 days on, 14 days off) and had a statistically significant deceleration in ALS Functional Rating Scale-Revised (ALSFRS-R) score decline compared to placebo; however, this was restricted to a narrowly targeted patient cohort [35].

Neither drug was effective at stopping or reversing the disease course. Supportive treatments tested in clinical studies, such as non-invasive ventilation, gastrostomy feeding, and interdisciplinary care, have not changed survival significantly but have invariably enhanced the quality of life, comfort, and functional ability in ALS [36].

## 3. CRISPR Technology

CRISPR technology represents a revolutionary approach to studying and potentially treating NDs by directly targeting underlying genetic alterations [37]. This gene editing tool has the potential to modulate gene expression, slow disease progression, and halt it entirely in some cases.

The application of CRISPR in NDs involves first mapping the genetic mutations associated with the disease and then designing CRISPR-Cas9 constructs to precisely target these mutations. Once engineered, CRISPR components, along with the Cas9 enzyme, which acts as a molecular scissor to cut DNA, are delivered into the brain using nanoparticles or viral vectors [38].

In vivo testing in animal models is crucial for evaluating the efficacy and safety of CRISPR-based therapies before they advance to human clinical trials. However, several challenges must be addressed, including efficient delivery methods, minimization of off-target effects, and ethical concerns related to gene manipulation [39].

**Figure 1:**
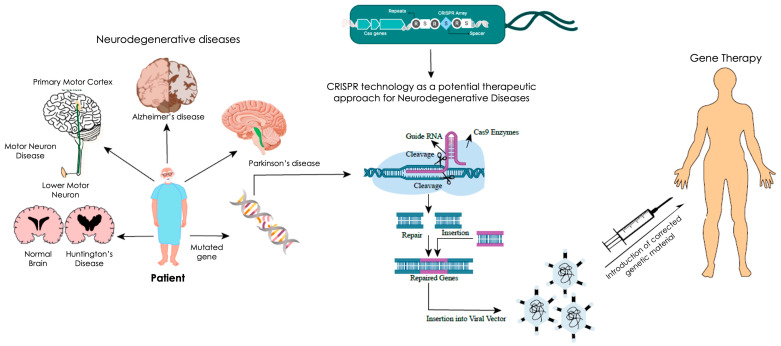
CRISPR Technology.

Despite these challenges, CRISPR technology has great potential to transform the therapeutic landscape of NDs, offering a paradigm shift from symptomatic treatment to disease-modifying interventions [6] (Figure 1).

### 3.1. Principle of CRISPR-Cas9 Technology

CRISPR is an innovative genome-editing technology that enables precise modification of DNA in living organisms [40]. The system comprises two key components: the CRISPR sequence and the Cas9 (CRISPR-associated protein 9) enzyme. The CRISPR sequence is a segment of DNA containing short, repetitive base sequences, originally discovered as a natural defense mechanism in single-celled microorganisms, such as bacteria and archaea. These organisms utilize CRISPR-derived RNA in complex with various Cas proteins, including the well-characterized thermonuclease-helicase effector protein Cas9, to target and eliminate viruses and other foreign genetic elements [41]. The CRISPR-Cas9 system operates by precisely cutting DNA at designated genomic sites. Fragments of viral DNA, known as spacers, are integrated into the CRISPR array and serve as a form of genetic memory for past infections. Cas9 is an endonuclease enzyme associated with the CRISPR sequence that functions as a molecular scissor and cleaves DNA at specific locations. The Cas9 enzyme is guided to its target site by a guide RNA (gRNA), which is designed to be complementary to the target DNA sequence. gRNA is a synthetic RNA molecule composed of two essential components: (i) crRNA (CRISPR RNA), complementary to the target DNA sequence, and (ii) tracrRNA (trans-activating crRNA), which forms a complex with the crRNA and recruits Cas9 for DNA cleavage [42]. The CRISPR-Cas9 complex binds to a 20-nucleotide DNA sequence known as the protospacer, which is immediately upstream of an NGG motif known as the protospacer-adjacent motif (PAM). The Cas9 enzyme introduces a double-strand break (DSB), precisely three base pairs upstream of the NGG motif, which initiates the editing process [43].

#### Mechanism of Cas9-Based Genome Editing Technology

Cas9 is an enzyme with two distinct nuclease domains, HNH and RuvC, that cleave target and non-target DNA strands, respectively [44]. Inactivation of either nuclease domain results in Cas9 nickase (nCas9), which is capable of cleaving only one DNA strand. Further inactivation of both nuclease domains generates dead Cas9 (dCas9), which retains the ability to bind to the target DNA but lacks enzymatic activity [45]. Notably, nCas9 has significant applications in base and prime editing, enabling precise genome modifications without requiring double-strand breaks (DSBs) [46]. Specifically, base editing enables precise, single-nucleotide changes in the DNA without creating double-stranded breaks (DSBs) or requiring donor DNA templates. Prime editing expands genome editing capability to include all types of point mutations, insertions, and deletions, with minimal DSBs and no donor templates.

Improving the editing efficiency of the CRISPR-Cas9 system is crucial for broader application. Several strategies have been developed to enhance CRISPR-Cas9 activity, including chromatin modification, which increases the accessibility of the target DNA [47], and the recruitment of end-processing enzymes to refine the precision of Cas9-induced edits [48]. and optimization of the gRNA structure, including increasing the duplex length and disrupting terminator sequences, which significantly improves the Cas9-mediated editing efficiency [49].

**Figure 2:**
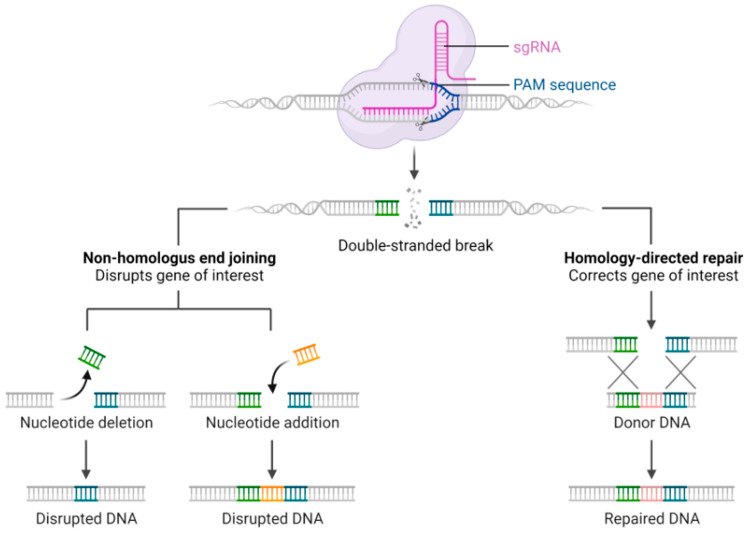
CRISPR genome editing strategy. At the top, the Cas9 protein, guided by a single guide RNA (sgRNA, shown in pink), binds to a complementary target DNA sequence adjacent to a PAM (Protospacer Adjacent Motif) sequence (shown in blue). Upon binding, Cas9 introduces a site-specific double-stranded break in the DNA. Following the DSB, the cell can repair the damage via two main pathways: (I) Non-Homologous End Joining (NHEJ): this error-prone repair mechanism ligates the broken DNA ends without a template, often resulting in small insertions or deletions (indels). These indels can disrupt the reading frame of the gene, leading to disrupted or nonfunctional DNA. The figure illustrates two outcomes: nucleotide deletion leading to disrupted DNA, and nucleotide addition (or random insertions), also resulting in disrupted DNA. (II) Homology-Directed Repair (HDR): this high-fidelity repair mechanism uses a homologous DNA template (donor DNA, shown in pink) to repair the break precisely. HDR enables the correction or replacement of a specific gene of interest, resulting in accurately repaired DNA.

Two major DNA repair pathways play critical roles in CRISPR-based genome editing. Non-Homologous End Joining (NHEJ) is characterized by small insertions or deletions (INDELs) at targeted genomic sites. This process often introduces frameshift mutations, effectively knocking out gene function. Homology-Directed Repair (HDR) is a precise DNA repair mechanism that facilitates the introduction of specific point mutations and the insertion or replacement of desired sequences at defined genomic locations [50] (Figure 2).

## 4. Genome Editing by Using CRISPR-Cas9 System in Neurodegenerative Diseases

CRISPR/Cas9 gene editing has emerged as a promising approach for targeting central nervous system (CNS) cells, including both neurons and glial cells, to address the underlying genetic causes of neurodegenerative diseases such as Alzheimer’s disease, Parkinson’s disease, and Huntington’s disease. The system enables precise modification or disruption of disease-associated genes in post-mitotic neurons and glia, which are otherwise challenging to manipulate due to their non-dividing nature and the restrictive blood-brain barrier [51,52,53,54,55,56].

Recent preclinical studies have demonstrated that CRISPR/Cas9 can be delivered to CNS cells using both viral (e.g., adeno-associated virus) and non-viral (e.g., nanocapsules, amphiphilic nanocomplexes) vectors. These delivery systems have achieved efficient in vivo genome editing in neurons, with minimal off-target effects and limited glial activation, as shown in mouse models of Alzheimer’s disease and Parkinson’s disease [6,7,8,9]. For example, targeting the *BACE1* gene in neurons reduced amyloid pathology and improved cognitive function in Alzheimer’s models, while editing α-synuclein in neuronal cells alleviated Parkinsonian toxicity [57,58,59,60,61,62,63,64,65,66,67,68,69].

CRISPR/Cas9 has also been used to generate more accurate disease models by editing genes in both neurons and glia, facilitating the study of disease mechanisms and the identification of therapeutic targets [52]. While most applications have focused on neurons, emerging strategies are exploring glial cell editing to modulate neuroinflammation and support neuronal survival [60]. Glial cells, including astrocytes, microglia, and oligodendrocytes, play crucial roles in maintaining CNS homeostasis and modulating disease progression. Emerging research has highlighted the contribution of glial dysfunction to the pathogenesis of various NDs, making these cells attractive targets for therapeutic intervention.

**Figure 3:**
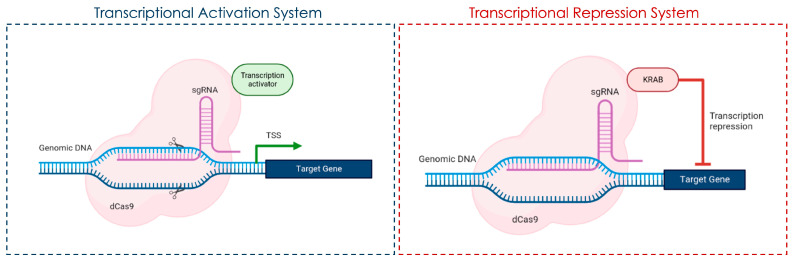
CRISPR-based gene expression regulation. The Transcriptional Activation System used dCas9 (a “dead” Cas9 lacking nuclease activity) guided by a single-guide RNA (sgRNA) to a specific DNA sequence near the promoter of a target gene. A transcriptional activator is fused to the dCas9 protein or recruited via the sgRNA scaffold. Finally, the dCas9-activator complex binds upstream of the transcription start site (TSS), facilitating increased transcription of the target gene. This approach is commonly used in CRISPRa (CRISPR activation) to upregulate gene expression without altering the genomic sequence. The transcriptional repression system, referred to as CRISPRi (CRISPR interference), uses dCas9 bound to a target sequence near the promoter region, guided by an sgRNA. In this case, dCas9 is fused to a KRAB (Krüppel-associated box) transcriptional repressor domain. The KRAB-dCas9 complex blocks transcription initiation or recruits chromatin-modifying factors that lead to gene silencing.

CRISPR-mediated gene knockout in microglia has been shown to modulate neuroinflammatory responses in models of Alzheimer’s disease. For example, targeted CRISPR/Cas9 editing of the glia maturation factor (*GMF*) gene in microglia led to reduced GMF expression and suppressed microglial activation, as evidenced by decreased p38 MAPK phosphorylation and proinflammatory cytokine production in vitro, supporting the potential for microglia-targeted gene editing to attenuate neuroinflammation in Alzheimer’s disease models [61]. Additionally, CRISPR interference (CRISPRi) and activation (CRISPRa) platforms in human iPSC-derived microglia have enabled systematic identification of genetic regulators of microglial activation and disease-associated states, including those relevant to neurodegeneration [62] (Figure 3). These approaches have uncovered genes that control microglial survival, activation, and phagocytosis, providing a foundation for therapeutic targeting in neurodegenerative diseases such as Alzheimer’s and ALS.

For astrocytes, CRISPR-based gene editing has been used to dissect and modulate pathways involved in glutamate transport, oxidative stress responses, and neurotrophic factor secretion. CRISPRi screens in human iPSC-derived astrocytes have identified regulators of distinct inflammatory reactive states, including pathways downstream of NF-κB and STAT3, which are implicated in neuroinflammation and neurodegeneration [63]. Reviews highlight that astrocytic glutamate transporters (GLT-1/EAAT2 and GLAST/EAAT1) are critical for glutamate homeostasis, and their dysregulation contributes to excitotoxicity in ALS and Alzheimer’s disease; while direct CRISPR-mediated upregulation of these transporters is a promising strategy, most published work to date has focused on pharmacological or transcriptional modulation [64,65]. CRISPR-based approaches are also being explored to enhance astrocytic neuroprotective functions, including antioxidant defense and the secretion of neurotrophic factors, as part of broader neuroprotective strategies [66].

Collectively, these studies demonstrate that CRISPR/Cas9 technology is a powerful tool for both mechanistic studies and the development of glia-targeted therapies, complementing neuron-focused approaches in neurodegenerative diseases. This is due to the central regulatory and supportive roles of glial cells in CNS homeostasis and pathology.

### 4.1. CRISPR/Cas9 in Alzheimer’s Disease

AD is the most prevalent cause of dementia and ultimately leads to mortality. It is characterized by the loss of cholinergic neurons, accumulation of intracellular neurofibrillary tangles composed of hyperphosphorylated tau protein [67], and the presence of extracellular amyloid-beta (Aβ) plaques in the brain. While both Aβ and Tau exhibit neurotoxicity independently, their interaction plays a significant role in disease progression, as Aβ accumulation can trigger tau hyperphosphorylation.

Over 300 mutations have been identified in the genes encoding amyloid precursor protein (*APP*), presenilin 1 (*PSEN1*), and presenilin 2 (*PSEN2*), all of which are associated with AD [68].

The CRISPR/Cas9 system holds great promise for modifying genes involved in Aβ production, such as APP and BACE1. BACE1 encodes β-secretase, which cleaves APP to generate Aβ peptides. By knocking out or reducing the expression of these genes, CRISPR/Cas9 can decrease amyloid plaque formation, thereby mitigating one of the primary pathological processes in AD [69].

Recent studies have demonstrated that CRISPR-Cas9-mediated correction of neurons derived from fibroblasts of individuals with the PSEN2N141I mutation can normalize the Aβ42/40 ratio and restore the electrophysiological deficits associated with the mutation [70].

Among the three common allelic variants of the apolipoprotein E (APOE) gene—ε2, ε3, and ε4—the ε4 allele is strongly associated with increased risk of late-onset, sporadic Alzheimer’s disease, while ε2 appears to be protective [70]. CRISPR/Cas9 can potentially convert the APOE ε4 allele into ε3 or ε2 forms, thereby reducing the risk or severity of AD. This gene-editing strategy offers the potential to prevent or delay disease onset in individuals with a genetic predisposition.

Wadhwani et al. demonstrated that neurons derived from iPSCs of patients carrying the APOE ε4/ε3 genotype exhibited reduced Aβ secretion and decreased tau phosphorylation when corrected to ε3/ε3 using CRISPR/Cas9-mediated overexpression of Cas9 nuclease [71].

The MAPT gene, which encodes tau protein, is another potential target for CRISPR/Cas9-based interventions. Modifying MAPT to reduce tau expression or prevent hyperphosphorylation may limit the formation of neurofibrillary tangles, thereby preserving neuronal function and slowing the disease progression.

Although CRISPR/Cas9 represents a promising and innovative approach to AD treatment, several critical challenges must be addressed before its clinical application. These include ensuring safety and efficacy through additional preclinical studies in appropriate models, developing efficient delivery systems capable of transporting CRISPR components across the blood-brain barrier and into target cells, and addressing ethical concerns related to genome editing, particularly regarding potential unintended consequences and long-term effects.

Although CRISPR/Cas9 offers promising strategies to modify key genes involved in AD pathogenesis, including *APP*, *BACE1*, *PSEN2*, *APOE*, and *MAPT*, its application remains largely experimental. Preclinical studies have demonstrated the feasibility of correcting pathogenic mutations, altering amyloid and tau pathology, and restoring neuronal function in cellular models. However, significant limitations persist. These include challenges in achieving precise, safe, and efficient delivery across the blood–brain barrier, potential off-target effects, and the need for long-term efficacy and safety validation in vivo.

### 4.2. CRISPR/Cas9 in Huntington’s Disease

Huntington’s disease (HD) is an autosomal dominant neurodegenerative disorder caused by a single mutation—specifically, an abnormal expansion of cytosine-adenine-guanine (CAG) repeats within the *huntingtin* (*HTT*) gene [72]. This mutation results in the production of a toxic form of the huntingtin protein, leading to progressive neuronal degeneration, especially in the striatum and cerebral cortex. Clinically, HD manifests as motor dysfunction, cognitive decline, and various psychiatric symptoms.

CRISPR/Cas9-based gene editing offers several promising therapeutic strategies for HD. One direct approach involves targeting the mutant *HTT* gene to excise or contract the expanded CAG repeats. Some studies have demonstrated that CRISPR/Cas9 can successfully remove the expanded repeat region, restoring the CAG tract to normal length and thereby reducing the production of the mutant protein [73].

A major area of focus is allele-specific editing, which aims to selectively inactivate or modify only the mutant *HTT* allele while preserving the normal allele. Since HD is caused by a single mutant copy of the gene, this selective approach reduces the risk of impairing the essential functions of the wild-type huntingtin protein. Researchers have developed guide RNAs (gRNAs) that target either the expanded CAG repeats or single-nucleotide polymorphisms (SNPs) unique to the mutant allele. For instance, Shin et al. designed an allele-specific CRISPR/Cas9 strategy that exploited PAM-altering SNPs (PASs) to selectively inactivate the mutant allele via nonsense-mediated decay (NMD), thereby silencing the production of the toxic protein [74].

In addition to gene editing, CRISPR/Cas9 can be used for gene silencing strategies that do not involve DNA cleavage. CRISPR interference (CRISPRi), which utilizes a catalytically inactive Cas9 (dCas9) fused to transcriptional repressors, can suppress transcription of the mutant allele. Seo et al. showed that CRISPRi effectively reduced mutant HTT expression while preserving the expression of the wild-type allele [75].

Another potential strategy is homology-directed repair (HDR), which pairs CRISPR/Cas9 with a DNA repair template to correct the expanded CAG repeats at the genomic level. While this approach offers the possibility of precise and permanent correction, it is important to note that HDR is significantly less efficient in non-dividing neurons than in dividing cells, limiting its feasibility in post-mitotic brain tissue [76]. Nonetheless, ongoing efforts to enhance HDR efficiency may eventually make this a viable therapeutic option.

In summary, CRISPR/Cas9 offers multiple avenues for HD treatment: excision of expanded repeats, allele-specific silencing or editing of the mutant allele, CRISPRi-mediated transcriptional repression, and template-driven gene correction via HDR. Although these strategies show substantial promise, further research is necessary to optimize editing efficiency, improve delivery to neural tissue, and ensure long-term safety prior to clinical application.

### 4.3. CRISPR/Cas9 in Parkinson’s Disease

PD is a widespread and progressive neurodegenerative disorder characterized by the selective loss of dopaminergic neurons (DNs) in the substantia nigra pars compacta (SNpc). This degeneration leads to a marked reduction in dopamine levels in the striatum, resulting in impaired motor circuit function. The cardinal clinical features of PD include resting tremor, bradykinesia, and muscular rigidity [77].

Genetic factors play a significant role in familial PD, with several genes identified as key contributors, including SNCA, PARK2, LRRK2, PINK1, GBA, DJ-1, UCHL1, and ATP13A2 [78]. Familial PD can follow either autosomal-dominant or autosomal-recessive inheritance patterns. Autosomal-recessive forms are commonly associated with mutations in PINK1, DJ-1 (PARK7), and PARK2, while mutations in LRRK2, PARK2, and SNCA are among the most frequently implicated genetic alterations in both familial and sporadic PD, contributing to its rising incidence and broader clinical recognition [79].

CRISPR-Cas9 genome editing has emerged as a powerful tool for modeling PD-related mutations. For example, one study employing CRISPR-Cas9 in combination with somatic cell nuclear transfer achieved a double knockout of PARK2 and PINK1 in domestic pig models, with a success rate of approximately 38%. Here, “success rate” refers to the proportion of edited colonies that were confirmed to be homozygous knockouts for both target genes [68].

In another study, researchers used CRISPR/Cas9 to delete PARKIN (PRKN), DJ-1 (PARK7), and ATP13A2 (PARK9) in isogenic lines of nigral dopaminergic neurons. Subsequent transcriptomic and proteomic analyses revealed dysregulation of oxidative stress pathways as a shared pathological mechanism across all edited lines [80]. These findings align with the known roles of the aforementioned genes in maintaining mitochondrial function and redox balance; for instance, mutations in PARK2 and PINK1 impair mitophagy, while DJ-1 is a known oxidative stress sensor.

CRISPR/Cas9 has thus proven invaluable for dissecting the molecular underpinnings of PD, particularly the contribution of oxidative stress to dopaminergic neuron degeneration. While its use in basic research is well established, therapeutic application remains in early development. Major challenges include the polygenic nature of PD, efficient and targeted delivery to affected brain regions, and minimization of off-target effects. Furthermore, given that most PD cases are sporadic, gene modulation strategies such as CRISPRi or antisense oligonucleotides targeting overexpressed genes such as SNCA may offer more feasible short-term therapeutic avenues.

In summary, CRISPR-based platforms offer powerful means to model and potentially correct genetic forms of PD. However, successful clinical translation will depend on overcoming significant hurdles related to delivery, safety, and disease heterogeneity.

### 4.4. CRISPR/Cas9 in Amyotrophic Lateral Sclerosis

Amyotrophic lateral sclerosis (ALS) is a progressive neurodegenerative disorder characterized by the loss of upper and lower motor neurons, leading to muscle weakness, paralysis, and ultimately respiratory failure within a few years. ALS is causally linked to mutations in key genes, including SOD1, C9orf72, FUS, and TARDBP, which drive neuronal dysfunction and degeneration through distinct pathological mechanisms. SOD1 mutations result in misfolded SOD1 protein aggregation, leading to oxidative stress and neuronal toxicity. C9orf72 repeat expansions have been associated with the formation of RNA foci and dipeptide repeat proteins (DPRs), which are hypothesized to contribute to cellular dysfunction, although the exact mechanisms of toxicity remain under investigation [81]. TARDBP mutations promote aberrant aggregation of TDP-43, interfering with RNA processing and contributing to neurodegeneration [82].

CRISPR-mediated gene editing has emerged as a promising therapeutic intervention to target ALS-associated genetic mutations at their source. Preclinical studies have demonstrated that CRISPR/Cas9-mediated gene knockdown can repress mutant SOD1 expression, resulting in reduced misfolded SOD1 protein, improved motor function, delayed disease onset, and extended survival in SOD1-ALS mouse models. These findings are supported by multiple studies using adeno-associated viral vectors to deliver CRISPR/Cas9 components, leading to significant reduction of mutant SOD1 in the central nervous system and amelioration of ALS phenotypes [83,84,85].

CRISPR/Cas9-mediated excision of pathogenic C9orf72 repeat expansions has been shown to prevent the formation of toxic RNA aggregates and dipeptide repeat proteins, restoring cellular function. In both in vitro and in vivo models, excision of the C9orf72 hexanucleotide repeat expansion using CRISPR/Cas9 reduced RNA foci and dipeptide repeat protein accumulation and rescued major disease mechanisms in patient-derived neurons and animal models. Additionally, RNA-targeting CRISPR systems (e.g., Cas13) have been shown to reduce both sense and antisense C9orf72 repeat RNAs and their associated dipeptide repeat proteins, further supporting the therapeutic potential of CRISPR-based approaches for C9orf72-linked ALS [86,87,88,89].

These studies collectively highlight the potential of CRISPR-mediated gene editing to directly target and mitigate the molecular pathology of ALS caused by SOD1 mutations and C9orf72 repeat expansions.

HDR-based CRISPR editing has been used to correct TARDBP mutations, reduce TDP-43 aggregation, and reestablish cellular homeostasis [90].

Preclinical studies have demonstrated the therapeutic potential of CRISPR-based approaches in the treatment of ALS. CRISPR/Cas9-mediated silencing of SOD1 in ALS animal models significantly improves motor function and extends survival [91].

CRISPR deletion of C9orf72 repeat expansions in iPSC-derived neurons from ALS patients successfully restored neuronal function, reduced RNA foci, and decreased the toxicity of dipeptide repeat proteins [92]. CRISPR correction of TARDBP mutations effectively reduced TDP-43 aggregation, a hallmark pathological feature of ALS.

In summary, ALS is driven by diverse genetic mutations and multifaceted pathological mechanisms, and CRISPR-based gene editing offers a promising therapeutic avenue for directly targeting disease-causing mutations and potentially restoring neuronal function. Ongoing research and clinical translation of these technologies are poised to transform the management of ALS, moving toward disease-modifying interventions for this currently incurable condition.

## 5. CRISPR-Mediated Gene Expression Modulation

CRISPR Interference (CRISPRi) is a powerful technique for gene repression that downregulates gene expression without inducing double-strand breaks in the DNA. This method relies on a catalytically inactive Cas9 (dCas9) protein fused to repressor domains and guided to the target gene by a specific guide RNA (gRNA). Once bound, CRISPRi reduces transcription by blocking RNA polymerase activity or by recruiting chromatin-modifying enzymes [93].

The CRISPRmod CRISPRi system consists of two key components: (i) a modified form of dCas9, which is conjugated to transcriptional repressor proteins (SALL1 and SDS3), and (ii) a specially designed gRNA that binds adjacent to the transcriptional start site (TSS) to guide the complex to the target gene [94].

After binding, the dCas9-SALL1-SDS3 complex interacts with gRNA, guiding the repressor complex to the target DNA site and effectively inhibiting gene transcription [95].

CRISPRi suppresses gene expression by either blocking transcription through direct binding of dCas9-repressor complexes to the gene locus or recruiting chromatin-modifying enzymes, which alter chromatin structure and further suppress transcription.

During this process, the dCas9 protein, when fused to a transcriptional repressor domain, binds to a specific DNA sequence, recruits chromatin-modifying enzymes, and effectively silences gene expression.

CRISPRi-based techniques have demonstrated broad applicability in bacterial systems, including bacteria [96], Streptococcus thermophilus [97], and mycobacteria [98].

CRISPRi has been modified and optimized for gene manipulation in bacterial engineering without concerns regarding its efficacy and reliability [99]. In addition to its bacterial applications, CRISPRi is a powerful tool for metabolic engineering and gene regulation in complex systems. Notably, it has been utilized in cyanobacteria as a precise metabolic engineering controller [100] and in human induced pluripotent stem cells (iPSCs), where it enables gene activation or repression to control cellular functions [101].

In conclusion, CRISPRi is a highly effective and versatile gene-silencing tool with broad applications in microbial and human cell systems. CRISPRi continues to revolutionize genetic engineering, functional genomics, and therapeutic research by precisely regulating gene expression without altering DNA sequences.

### CRISPR Activation (CRSIPRa)

The current landscape of CRISPR activation (CRISPRa) systems can be categorized into two primary approaches: (i) the cis-activation system, which involves the direct fusion of a catalytically inactive Cas9 (dCas9) with a transcriptional activation domain and (ii) the trans-activation system, which uses adaptor proteins or modified guide RNAs (sgRNAs) to recruit transcriptional activators either to the dCas9 structure or to the sgRNA scaffold.

The first CRISPRa system was developed by fusing VP44 or VP48 with dSpCas9, a modified version of Cas9 [102]. These studies demonstrate that the positioning of dCas9 at transcription start sites (TSSs) plays a key role in determining whether transcription is activated or inhibited, as it interferes with the initiation of the transcriptional process. Additionally, CRISPRa achieves high activation efficiency when dCas9 is linked with transcriptional activators, allowing it to precisely target gene promoters.

The simplest CRISPRa system consists of dCas9 fused to a single activator such as VP64. However, more complex activation strategies have been developed, including (i) co-expression systems, such as epitope-tagged dCas9 with antibody-stimulated effector proteins; (ii) fusion-based approaches, such as SunTag, which amplifies transcriptional activation through multiple activating domains (e.g., dCas9-VPR); and (iii) modified sgRNA scaffolds, which enhance gene activation by recruiting additional activators (e.g., dCas9-VP64 with modified sgRNA scaffolds).

Unlike Cas9 or Cas9 nickase, which induce permanent genomic modifications, CRISPRa-based gene activation does not irreversibly alter the DNA sequences. This makes CRISPRa a valuable tool for studying gene regulation in both mouse and human cells without introducing permanent genetic changes.

The efficacy of CRISPRa in ex vivo models has been demonstrated in various cell types ranging from bacteria to mammalian cells and plants. One of the most promising applications of CRISPRa is in disease modeling using animal models. The CRISPR-Cas system has been used to genetically engineer rabbit models to study complex diseases, including congenital cataracts, Duchenne muscular dystrophy (DMD), and X-linked hypophosphatemia (XLH). These models have significantly improved our understanding of disease mechanisms, thereby facilitating the development of effective therapeutic interventions [103].

Beyond its role in animal models, CRISPRa has also been successfully applied to gene activation in various cellular systems, such as induced pluripotent stem cells (iPSCs), further demonstrating its versatility [104].

CRISPRa has shown great promise in activating genes involved in NDs, highlighting its flexibility as a genetic research tool. By enabling precise gene regulation, CRISPRa is an invaluable approach for studying gene function and developing therapeutic strategies for genetic disorders [105].

CRISPRa is a powerful and versatile tool for genetic research and therapeutic application. Its ability to activate gene expression across diverse biological systems, including human cells, animal models, and plants, reinforces its potential for advancing biomedical research and precision medicine.

## 6. Preclinical Studies: Gene Expression Modulation for Therapeutic Benefits

Konstantinidis et al. demonstrated that the CRISPR-Cas9 Type II system derived from Streptococcus pyogenes can precisely target the PSEN1M146L allele in human fibroblasts. Their study highlighted the critical role of the APOE genotype in both familial and sporadic forms of AD [106].

A study conducted by Arango et al. [86] revealed that CRISPR-Cas9-mediated gene editing successfully excised CAG repeats associated with Huntington’s disease (HD) using a mouse model. This research supports the potential of CRISPR technology to target the genetic mutations underlying neurodegenerative disorders.

The *brain-derived neurotrophic factor* (*BDNF*) gene is a potential target for enhancing neuronal function. In neurons derived from human iPSCs, CRISPRa was used to upregulate BDNF expression in neurons derived from human iPSCs, demonstrating its therapeutic potential in neurodegeneration [107].

Recent in vitro studies suggested that both CRISPRi and CRISPRa can influence gene expression and potentially ameliorate neurological disorders. For instance, CRISPRi has been employed to knock down the expression of amyloid precursor protein (*APP*), a key factor in Alzheimer’s disease pathology. This was achieved in pluripotent stem cells derived from human neurons carrying PSEN1 mutations across multiple induced pluripotent stem cell (iPSC) lines. The first clinical trial utilizing iPSC-derived cells for the treatment of PD was conducted in August 2018 at the Kyoto University Hospital, Japan. In this trial, patients with PD received injections of dopaminergic progenitor cells derived from clinical-grade human iPSCs. These cells, having undergone extensive quality control screenings, were subsequently transplanted into the bilateral putamen to restore the lost dopaminergic neurons [6]. Additionally, in a mouse model of Parkinson’s, CRISPR/Cas9-mediated gene editing was used to introduce a mutation in the *α-synuclein* gene, a gene closely linked to PD. Although this approach successfully reduced α-synuclein aggregation, it did not lead to significant improvements in motor function, suggesting the need for further research into CRISPR-based therapies for PD. Another study using CRISPR/Cas9 technology in a mouse model of AD successfully corrected the amyloid precursor protein (*APP*) gene, which is a major factor in Aβ toxicity. These findings revealed a reduction in amyloid-β 42/40 levels, which are key biomarkers associated with the onset of AD. These results suggest that gene editing could serve as a promising approach for mitigating Alzheimer’s pathology [108,109].

CRISPR-based gene editing technologies have revolutionized the field of neurodegenerative disease research, from gene knockdown and activation to clinical applications in PD and AD. These studies highlight the transformative potential of CRISPR in understanding and treating neurodegenerative disorders, from gene knockdown and activation to clinical applications in PD and AD. However, further research is required to enhance therapeutic efficacy, optimize delivery methods, and address long-term safety concerns. There are no clinical trials available to study CRISPR therapeutics, specifically in patients with neurodegeneration. Research to date is still primarily in the preclinical stages based on laboratory and animal models to assess safety and efficacy before progressing towards human trials [37].

## 7. Comparison of Conventional Therapies and CRISPR Based Interventions

Conventional treatments for neurodegenerative disorders are symptom-palliative and treat downstream consequences such as neurotransmitter imbalance or motor deficits. They consist of medications such as levodopa, donepezil, and riluzole, which can offer relief but are unable to halt the course of the disease and have an elevated risk of adverse effects [10,29]. In contrast, CRISPR-based therapies target gene mutations responsible for ailments such as Alzheimer’s, Parkinson’s, Huntington’s, and ALS. CRISPR holds the promise of long-term and possibly curative effects through its ability to precisely edit the genome. Off-target effects, delivery across the blood-brain barrier, and ethical implications are issues that need to be resolved before clinical translation can become a widely available option [6]. Table 1 presents a comparison between CRISPR and current therapies for NDs. When applied to complex organisms, CRISPR has the potential to revolutionize medical treatment, agriculture, and various scientific fields by enabling precise modification of DNA sequences [109]. However, its use also introduces a minor ethical concern and challenges [110].

## 8. Limitations of Therapies of Neurodegenerative Diseases

Current therapies primarily focus on symptom management rather than disease modification. Many pharmacological treatments have significant side effects that limit their long-term use (e.g., dyskinesia in Parkinson’s patients treated with levodopa or cognitive side effects of ALS medications such as riluzole). Patients often discontinue treatment owing to adverse reactions. Physical therapy, occupational therapy, and cognitive rehabilitation improve the quality of life but do not alter disease progression [111].

Alternative therapies such as dietary interventions and neuroprotective lifestyle modifications have not been conclusively proven to slow disease progression. Finally, the blood-brain barrier prevents many therapeutic agents from reaching the brain at adequate concentrations [112]. Thus, the current therapies for NDs have significant limitations in terms of efficacy, accessibility, and disease modification.

In recent years, antisense oligonucleotide (ASO) technology has emerged as a transformative therapeutic strategy for several neurodegenerative diseases, with notable success in clinical applications [113]. ASOs are short, synthetic strands of nucleotides designed to bind RNA transcripts in a sequence-specific manner, thereby modulating gene expression through mechanisms such as splicing correction, transcript degradation, or translation inhibition.

A landmark example is the treatment of SMA with nusinersen (Spinraza), the first FDA-approved ASO therapy for a neurodegenerative condition [114]. Nusinersen modulates splicing of the *SMN2* gene to promote the inclusion of exon 7, thereby restoring the production of functional survival motor neuron (SMN) protein. Clinical trials have demonstrated significant improvements in motor function and survival, fundamentally changing the prognosis for SMA patients [113].

In ALS, ASO-based therapies have shown promise, particularly in patients with mutations in the SOD1 gene. A notable example is tofersen, an ASO that targets SOD1 mRNA to reduce toxic protein accumulation. Clinical studies, including a randomized, placebo-controlled trial published in the New England Journal of Medicine by Miller et al. [115], have demonstrated that repeated intrathecal injections of tofersen result in sustained reductions in SOD1 protein levels and a slower decline in functional status among patients with SOD1-ALS. Additional ASO therapies are under investigation for other genetically defined forms of ALS, such as those involving FUS and C9orf72 mutations, and are also being explored in Huntington’s disease, Alzheimer’s disease, and frontotemporal dementia. These efforts underscore the versatility of ASOs and their increasing clinical relevance.

Compared to CRISPR/Cas9-based genome editing, which is still largely in the preclinical or early clinical trial phase, ASOs represent a more established and rapidly deployable therapeutic platform. They also offer the advantage of reversibility and dose titration, which is particularly beneficial in the context of the central nervous system, where permanent genetic changes carry inherent risks.

Despite its revolutionary potential, CRISPR is far from having its important limitations and risks critically addressed before it can be clinically applied. Among these is the risk of off-target effects, where undesired DNA sequences are altered, potentially resulting in deleterious mutations. Another significant problem is the safe and effective delivery of CRISPR reagents to the brain. Although viral vectors and nanoparticles are widely used, they have limitations in terms of payload size capacity, immunogenicity, tissue specificity, and scalability. The irreversibility of gene editing also raises concerns regarding long-term safety when editing is performed in post-mitotic cells, such as neurons. Furthermore, ethical and regulatory considerations, especially regarding germline editing and equitable access, remain unresolved and may affect the pace of clinical translation. Therefore, while CRISPR holds promise as a disease-modifying intervention for neurodegenerative diseases, it must be approached with rigorous safety assessments, cautious optimism, and strong ethical oversight [116,117].

## 9. Delivery of CRISPR/Cas Components to the Central Nervous System

The successful application of CRISPR/Cas systems for treating neurological diseases hinges not only on precise gene targeting but also on the development of efficient, safe, and scalable delivery methods to the CNS. Delivering gene-editing components, especially Cas nucleases and guide RNAs (gRNAs), across the BBB and achieving widespread distribution within the brain remain significant challenges. This paragraph explores the primary delivery vectors and strategies currently under investigation.

### 9.1. Viral Vectors

#### 9.1.1. Adeno-Associated Virus (AAV)

AAV vectors are among the most widely used delivery platforms for CRISPR/Cas in vivo, particularly for CNS applications, due to their low immunogenicity and ability to transduce post-mitotic neurons. Certain serotypes (e.g., AAV9, AAV-PHP.B) show enhanced tropism for the CNS following systemic or intrathecal delivery [118,119].

However, AAV has a limited packaging capacity (~4.7 kb), which presents a major constraint when delivering the large *Streptococcus pyogenes Cas9* (*SpCas9*) gene (~4.2 kb) along with necessary regulatory elements and gRNA cassettes. To overcome this, several strategies have been developed, including the use of smaller Cas variants such as SaCas9 (Staphylococcus aureus Cas9, ~3.2 kb); split-Cas9 systems, where the *Cas9* gene is divided and delivered via dual AAV vectors and reconstituted in cells through trans-splicing or protein fragment complementation; and co-delivery of Cas9 and gRNAs via separate AAVs, though this may reduce efficiency [118,119,120].

#### 9.1.2. Lentiviral Vectors

Lentiviruses have higher packaging capacities (~8–10 kb) and the ability to integrate into the host genome, allowing for long-term expression of CRISPR components. They have been used to deliver CRISPR/Cas systems in animal models of neurodegeneration. However, their integrating nature poses safety concerns due to potential insertional mutagenesis. Lentiviral delivery is also less efficient in non-dividing neurons compared to AAV. These points are supported by recent reviews and primary research in the medical literature, which detail the advantages, limitations, and engineering strategies for both AAV and lentiviral vectors in CRISPR/Cas delivery for CNS and other in vivo applications [121].

### 9.2. Non-Viral Delivery Systems

#### 9.2.1. Liposomes and Lipid Nanoparticles (LNPs)

Lipid-based carriers such as liposomes and LNPs can encapsulate Cas9 mRNA or protein along with gRNA, allowing transient and non-integrating delivery. These platforms are associated with reduced immunogenicity and a lower risk of genome integration compared to viral vectors, as the Cas9 protein or mRNA is rapidly degraded after delivery, minimizing persistent expression and off-target effects [122]. LNPs have demonstrated clinical safety in non-CNS applications (e.g., mRNA vaccines, liver-targeted gene editing), but efficient delivery to the CNS remains a major challenge due to the BBB [123]. Without additional modifications, such as targeting ligands or physical BBB disruption (e.g., focused ultrasound), LNPs do not efficiently cross the BBB after systemic administration. Recent advances include the development of brain-targeting lipid formulations and intrathecal administration, which have shown improved CNS delivery and genome editing in preclinical models.

#### 9.2.2. Gold Nanoparticles, Exosomes, and Polymeric Nanocarriers

Gold nanoparticles, exosomes, and polymeric nanocarriers are indeed recognized as emerging platforms for CNS-targeted gene delivery. Gold nanoparticles have demonstrated the ability to transport oligonucleotides across brain endothelial cells and facilitate BBB penetration, especially when functionalized or combined with targeting ligands or exosome membranes, enhancing neuron-specific delivery and CNS accumulation in preclinical models. Exosomes, as natural extracellular vesicles, are increasingly engineered for brain-targeted delivery of nucleic acids due to their intrinsic biocompatibility, ability to cross the BBB, and potential for surface modification to improve neuron-specific targeting; however, challenges remain regarding standardization, scalability, and in vivo efficacy. Polymeric nanocarriers, including biodegradable nanoparticles, dendrimers, and nanogels, are also under active investigation for their capacity to protect genetic cargo and facilitate BBB transcytosis, with surface functionalization strategies being developed to enhance CNS targeting and delivery efficiency [124,125,126,127,128,129].

Despite these advances, the translation of these platforms to clinical application is limited by issues such as manufacturing scalability, reproducibility, and the need for robust demonstration of in vivo efficacy and safety in humans [130]. Collectively, the current medical literature supports the statement that these nanotechnologies offer novel, customizable approaches for CNS gene delivery but emphasizes that further research is required to address translational barriers and optimize clinical utility [130].

### 9.3. Routes of Administration

#### 9.3.1. Intrathecal Injection

Intrathecal delivery, involving direct injection into the CSF, enables broad CNS distribution of therapeutic agents [131,132]. Contrary to our previous wording, we now acknowledge that intrathecal injections are clinically viable for repeated use, as demonstrated by monthly ASO administration in SMA patients, where this route has shown both efficacy and tolerability [133]. CRISPR delivery via intrathecal injection is being actively explored, particularly in combination with AAV and LNPs [134].

#### 9.3.2. Intracerebral and Intravenous Routes

Direct stereotactic injection into specific brain regions provides localized delivery but is invasive and limits distribution. In contrast, intravenous delivery of BBB-penetrant AAV serotypes or modified nanoparticles holds promise for non-invasive, widespread CNS gene editing, though further improvements in targeting efficiency are needed [122,135].

Each delivery strategy presents trade-offs in terms of cargo capacity, transduction efficiency, duration of expression, cell-type specificity, and immunogenicity. Moreover, immune responses against Cas proteins or viral vectors, especially in repeat dosing scenarios, remain a major concern in clinical translation [136]. The optimal delivery method will likely vary depending on disease context, patient age, and target gene.

Efficient and safe delivery of CRISPR components to the CNS is a prerequisite for realizing gene editing as a viable therapeutic modality for neurodegenerative diseases. While viral vectors, particularly AAVs, remain the front-runners, non-viral systems are gaining momentum [137]. Continued innovation in vector design, delivery routes, and dosing strategies will be key to overcoming current limitations and ensuring broad, safe, and effective genome editing within the human brain.

## 10. Challenges in Drug Delivery and Ethical Considerations

Effective treatment of neurodegenerative diseases is increasingly limited by the difficulty in delivering therapeutic agents to the CNS. One key challenge is the poor delivery of therapeutic drugs to the CNS. This is mostly due to the blood-brain barrier, a highly regulated and specific interface that prevents the passage of most drugs and even large molecules, such as antibodies, enzymes, and gene editing tools. Therefore, it is very challenging to achieve therapeutic drug levels in the brain tissue, especially in areas of neurodegeneration.

Most drugs have short half-lives and low bioavailability and cause off-target effects because they are administered systemically [138]. Dopaminergic drugs for Parkinson’s, such as levodopa, experience decreased effectiveness over time through breakdown in the periphery and irregular delivery to the brain [94]. Likewise, monoclonal antibodies in the treatment of Alzheimer’s disease, such as lecanemab, must be administered intravenously and present off-target risks, such as Amyloid-Related Imaging Abnormalities (ARIAs), an indicator of poor specificity in targeting the CNS [139].

CRISPR-Cas9 technology represents a major advancement in the field of genetic engineering, offering high precision and versatility for targeted genome modification. However, its use also introduces a minor ethical concern and challenges. Delivery challenges have become even more critical for new therapies such as gene editing and gene silencing reagents. They also tend to require viral vectors or nanoparticle platforms for CNS delivery, both of which pose challenges in the areas of efficiency, scalability, immunogenicity, and tissue specificity. Targeting specific brain regions continues to be a key challenge, which carries the risk of inducing immune responses and off-target effects on non-neuronal tissues [140].

Although more specific, intracerebral or intrathecal injections are invasive and unsuitable for regular use. Accordingly, non-invasive efficient, and cell-targeted delivery systems may be the greatest obstacle for the bench-to-bedside translation of next-generation therapies, including CRISPR interventions.

Limited long-term safety data are available for these delivery systems, particularly for genome integration, immune activation, and off-target tissue toxicity. Accordingly, although molecular therapies boost excitement, the absence of efficient, safe, and targeted delivery systems remains a bottleneck for their clinical application in the clinic.

Although CRISPR-Cas9 is highly accurate, it is not immune to off-target effects, where unintended genetic modifications may occur [89]. This is because the target DNA sequence may share similarities with the other sequences within the genome. Such unintended changes could disrupt essential genes, leading to unpredictable consequences and increased health risks for individuals undergoing CRISPR-based interventions.

The ethical implications of off-target effects extend beyond those of the directly treated individuals. The heritable nature of genetic modifications raises concerns regarding their long-term impact on future generations. This aspect of CRISPR-based genome editing has sparked philosophical and ethical debate regarding the potential consequences of altering the human gene pool. To address these ethical concerns, CRISPR-Cas9 should be regulated within strict ethical and legal frameworks. Regulatory bodies play a crucial role in ensuring that CRISPR-based therapies undergo rigorous clinical trials to assess their safety and efficacy before receiving clinical approval [141].

Effective regulatory oversight should include comprehensive screening techniques to detect off-target effects, long-term monitoring regimens to assess the impact of genomic modifications over time, and ethical guidelines to balance scientific progress with societal welfare.

By adhering to stringent regulatory norms, stakeholders can mitigate ethical dilemmas and ensure that CRISPR technology is developed and applied responsibly.

The fundamental principle behind ethical genome editing is that it must be transparent and consensual. Individuals undergoing CRISPR-based therapy must be fully informed of the risks, benefits, and potential off-target effects of these interventions. Informed consent plays a critical role in protecting patient autonomy, ensuring that individuals have control over their genetic information and can make well-informed decisions regarding their treatment [142,143].

Another question to be addressed is that CRISPR/Cas9 gene editing in the brain may cause unwanted germline modifications due to the potential for off-target activity or unintended delivery of editing components to germ cells. While the primary intent of CNS-directed CRISPR therapies is somatic editing, in vivo delivery systems (such as viral vectors or nanoparticles) can theoretically reach gonadal tissue, raising the risk of germline modification. This risk is not unique to CNS applications but is inherent to most in vivo CRISPR-based therapeutics, as highlighted in recent reviews of CNS gene therapy development [122].

Animal data support the plausibility of this risk. In zebrafish, CRISPR-Cas9 editing of fertilized eggs resulted in large structural variants at both on-target and off-target sites, with 26% of offspring carrying an off-target mutation and 9% carrying a structural variant, demonstrating that unintended edits can be transmitted through the germline [143]. In mouse models, CRISPR-Cas9 editing in embryos has been shown to induce karyotype aberrations, including whole chromosome loss and genomic instability, which can propagate through embryonic development and potentially affect the germline. These findings underscore the need for rigorous preclinical assessment of off-target and germline effects, as well as the development of delivery strategies that minimize exposure to germ cells.

Current consensus in the medical literature is that while the risk of germline modification from CNS-targeted CRISPR/Cas9 is theoretical, it is a significant ethical and safety concern that must be quantified and mitigated in preclinical and clinical development [144,145,146].

Continued scientific research is essential to improve our understanding of off-target effects and develop strategies to minimize them. As CRISPR-based genome editing expands to various cell types, tissues, and organs, efforts must focus on enhancing the specificity of CRISPR editing strategies, developing more efficient delivery methods to precisely target genetic material, and innovating new gene-editing technologies that improve CRISPR-Cas9.

By refining CRISPR-mediated genome editing, researchers can identify why and how off-target effects occur and devise effective solutions to reduce these risks. This will ensure that CRISPR is safe and effective for treating genetic disorders.

In conclusion, CRISPR-Cas9 holds tremendous promise for treating genetic disorders and for advancing biomedical research. However, ethical challenges must be addressed carefully. Through rigorous regulation, transparency, and informed consent, CRISPR technology can be responsibly developed.

By prioritizing ethical frameworks, we can harness the potential of CRISPR while minimizing risks, paving the way for a future in which genetic editing can improve human health and well-being in a responsible and ethical manner.

## 11. Conclusions

CRISPR technology has emerged as a highly versatile and precise tool for genome editing, revolutionizing biomedical research and therapeutic applications. The CRISPR/Cas9 system offers several advantages over conventional gene-editing methods, providing unmatched precision and efficiency in disease treatment.

Ongoing research and technological advancements in CRISPR-based therapies have tremendous potential to transform the therapeutic landscape of age-related diseases.

The accumulation of abnormal proteins and peptides in the CNS is a hallmark of many neurodegenerative disorders, including AD and PD. In response to these challenges, gene therapy has emerged as a promising strategy for correcting genetic mutations associated with neurodegenerative diseases and modulating the expression of disease-associated proteins to prevent or slow disease progression.

Despite its potential, CRISPR/Cas9-based genome editing poses significant concerns, particularly regarding off-target effects, which may lead to unintended genetic modifications and unexpected insertions and deletions (INDELs) that can occur during DNA repair following CRISPR/Cas9-mediated cleavage.

These challenges highlight the need for continuous advancements in research and technology in order to enhance the precision, safety, and efficacy of CRISPR-based interventions.

CRISPR technology represents a groundbreaking innovation in gene therapy, offering the potential to revolutionize the treatment of neurodegenerative and age-related diseases. However, further refinement of gene-editing techniques is necessary to minimize off-target effects and improve precision. With ongoing scientific advancements, CRISPR holds great promise for shaping the future of precision medicine and targeted therapy.

## Figures and Tables

**Table 1:** Comparison between current therapeutic medication and CRISPR-based Interventions.

	Current Therapies	CRISPR-Based Interventions
Primary Goal	Symptomatic relief, slow down the progression of diseases	Target the genetic causes to reverse the disease progression
Approach	Medications, physical therapy, symptom management	Gene editing, targeted genetic modifications
Target	Symptoms such as motor dysfunction and cognitive decline	Specific genes responsible for neurodegenerative conditions
Personalization	No. It is a generalized treatment for patient groups	Personalized medicine tailored to individual genetic profiles
Efficacy	Alleviates symptoms, limited in halting or reversing progression	Potentially high efficacy in correcting genetic mutations and halting progression
Onset of Action	Immediate symptomatic relief	Long-term genetic correction, may take time to show effects
Quality of Life	Improves daily functioning and overall well-being	Potentially significant improvements by addressing root causes
Side Effects	Possible side effects from medications, e.g., increase in disease progression	Risks of off-target effects and immune responses
Research and Development	Well-established, with decades of clinical data	Rapidly advancing, with several preclinical and some clinical studies
Current Availability	Widely available and commonly used	Experimental, with limited clinical availability

## Data Availability

No new data were created or analyzed in this study. Data sharing is not applicable to this article.
